# The adhesion G protein-coupled receptor GPR56 is a cell-autonomous regulator of oligodendrocyte development

**DOI:** 10.1038/ncomms7121

**Published:** 2015-01-21

**Authors:** Stefanie Giera, Yiyu Deng, Rong Luo, Sarah D. Ackerman, Amit Mogha, Kelly R. Monk, Yanqin Ying, Sung-Jin Jeong, Manabu Makinodan, Allison R. Bialas, Bernard S. Chang, Beth Stevens, Gabriel Corfas, Xianhua Piao

**Affiliations:** 1 Division of Newborn Medicine, Department of Medicine, Boston Children’s Hospital and Harvard Medical School, Boston, Massachusetts 02115, USA; 2 Department of Developmental Biology, Washington University School of Medicine, St Louis, Missouri 63110, USA; 3 Hope Center for Neurological Disorders, Washington University School of Medicine, St Louis, Missouri 63110, USA; 4 F.M. Kirby Neurobiology Center, Children’s Hospital, Harvard Medical School, Boston, Massachusetts 02115, USA; 5 Department of Neurology, Harvard Medical School, Boston, Massachusetts 02115, USA; 6 Comprehensive Epilepsy Center, Department of Neurology, Beth Israel Deaconess Medical Center and Harvard Medical School, Boston, Massachusetts 02115, USA

## Abstract

Mutations in *GPR56*, a member of the adhesion G protein-coupled receptor family, cause a human brain malformation called bilateral frontoparietal polymicrogyria (BFPP). Magnetic resonance imaging (MRI) of BFPP brains reveals myelination defects in addition to brain malformation. However, the cellular role of GPR56 in oligodendrocyte development remains unknown. Here, we demonstrate that loss of *Gpr56* leads to hypomyelination of the central nervous system in mice. GPR56 levels are abundant throughout early stages of oligodendrocyte development, but are downregulated in myelinating oligodendrocytes. *Gpr56*-knockout mice manifest with decreased oligodendrocyte precursor cell (OPC) proliferation and diminished levels of active RhoA, leading to fewer mature oligodendrocytes and a reduced number of myelinated axons in the corpus callosum and optic nerves. Conditional ablation of *Gpr56* in OPCs leads to a reduced number of mature oligodendrocytes as seen in constitutive knockout of *Gpr56*. Together, our data define GPR56 as a cell-autonomous regulator of oligodendrocyte development.

## Abstract (web summary)

Mutations in GPR56, a member of the adhesion G protein-coupled receptor family, cause a specific human brain malformation and myelination defects but the cellular causes remain unclear. Here the authors show that loss of *Gpr56* in mice leads to decreased oligodendrocyte precursor cell proliferation and diminished levels of active RhoA.

## Graphical abstract

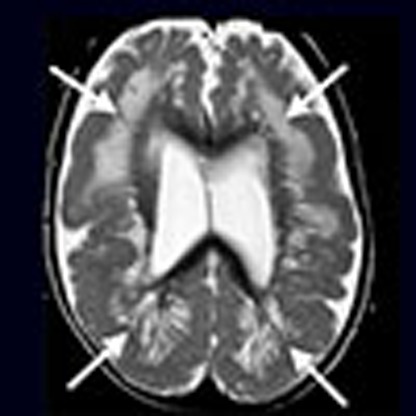

Myelin, the multilayered glial membrane surrounding axons, is paramount to axon conductivity and health in the vertebrate nervous system. In the central nervous system (CNS), myelin is formed by specialized glial cells called oligodendrocytes (OLs)^1^,^2^. The molecular mechanisms that govern OL development are only beginning to be elucidated, yet several studies have implicated extracellular matrix (ECM) proteins and their receptors as important extrinsic regulators of these processes^3^,^4^,^5^,^6^. The family of adhesion G protein-coupled receptors (aGPCRs) is a major class of ECM receptors that mediates cell–matrix interactions, and recent reports implicate aGPCRs as important regulators of myelination^7^,^8^,^9^,^10^,^11^. Thus, we hypothesize that these receptors could play a major role during OL development and myelination.

Mutations in *GPR56*, a member of the aGPCR family, cause a devastating human brain malformation called bilateral frontoparietal polymicrogyria (BFPP), in which the normal convoluted brain surface is replaced by numerous small gyri^12^,^13^. In addition to this cortical defect, BFPP brains also show signs of myelination abnormalities, namely reduced white matter volume and signal changes on MRIs^12^,^13^, suggesting that GPR56 is important for myelination. However, it is not clear whether this myelination abnormality results from abnormal neuronal development, an intrinsic defect in OLs, or both.

Previous biochemical studies demonstrate that BFPP-associated mutations result in loss of GPR56 function attributable to aberrant processing/trafficking of the protein or loss of its ligand binding ability^14^,^15^,^16^,^17^,^18^. Consistent with these findings, *Gpr56*-knockout mice exhibit forebrain and cerebellar defects similar to those seen in BFPP^19^,^20^. However, the cellular roles of GPR56 in CNS myelination remain unclear. A recent study shows that GPR56 regulates neural stem cell proliferation^21^, but its role in OL development remains unknown.

In light of the severe CNS myelination abnormalities seen in BFPP patients, we studied the cerebral white matter of *Gpr56*-knockout mice. We used histological, cellular and molecular approaches to characterize the defects caused by loss of GPR56. Here, we show that GPR56 levels are the highest in OL precursor cells (OPCs), and that GPR56 levels decline as OLs develop. We demonstrate that GPR56 is required for proper CNS myelination by controlling OPC proliferation, and that this is likely mediated by RhoA activation. Finally, we demonstrate that the defects caused by loss of GPR56 arise from a cell-autonomous defect in OL-lineage cells. Together, these studies define GPR56 as a novel regulator of OL development.

## Results

### Loss of GPR56 results in CNS myelination defects

**Figure 1:**
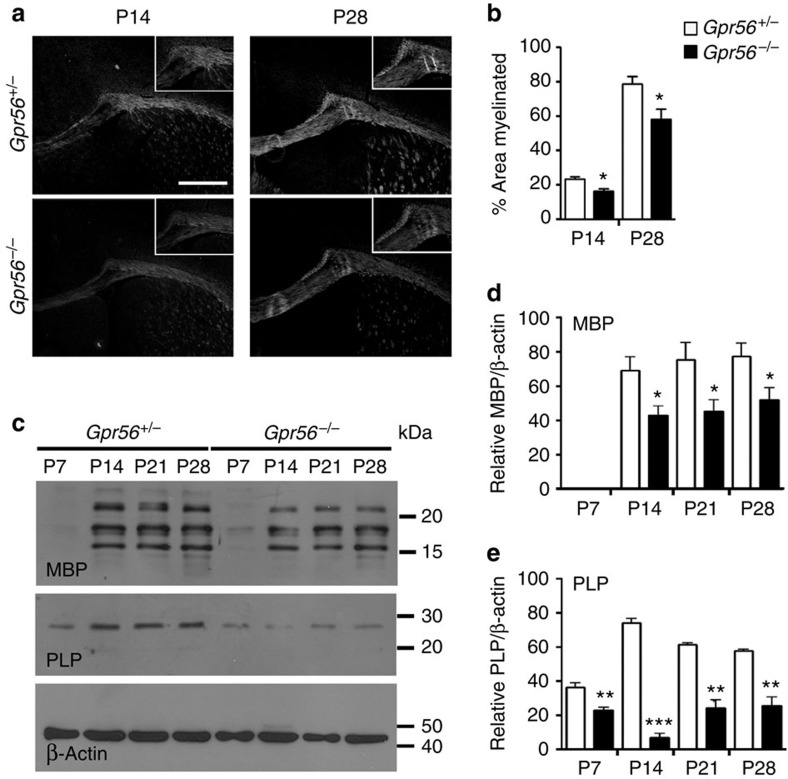
Loss of GPR56 causes CNS hypomyelination in mice. (**a**) Reduced FluoroMyelin staining was observed in the CC of P14 and P28 brains of *Gpr56*^−/−^ mice (lower panel) compared with their littermate control (upper panel). Scale bar, 500 μm. (**b**) Bar graphs depicted percentage of area myelinated. Myelin is reduced at P14 (*P=0.0144; unpaired *t*-test, *n*=4 per genotype) and P28 (*P=0.0499; unpaired *t*-test, *n*=3 per genotype) in the CC of *Gpr56*^−/−^ compared with controls. (**c**) Western blot analyses of MBP and PLP expression in the CC of *Gpr56*^+/−^ and *Gpr56*^−/−^ mice at P7-28. Loading control: β-actin. (**d**,**e**) Bar graphs depicted relative optical density of MBP and PLP to the loading control β-actin. MBP protein was significantly reduced in the CC of *Gpr56*^−/−^ compared with *Gpr56*^+/−^ littermates on P14 (*P=0.0285), P21 (*P=0.0315) and P28 (*P=0.0443). Unpaired *t*-test, *n*=3 per genotype. PLP protein was significantly decreased in the CC of *Gpr56*^−/−^ compared with *Gpr56*^+/−^ littermates on P7 (**P=0.0091), P14 (***P<0.0001), P21 (**P=0.0011) and P28 (**P=0.003). Unpaired *t*-test, *n*=4 per genotype. Error bars are means ± s.e.m.

CNS white matter abnormalities, manifesting as bilateral foci of T2 signal intensity change within the cerebral white matter on brain MRIs (Supplementary Fig. 1, right panel), is one of the hallmarks of BFPP brains^12^,^13^,^22^,^23^. To begin to investigate the role of GPR56 in CNS myelination, we examined the myelination status of *Gpr56*-knockout mouse brains^20^,^24^. We have previously shown that there is no brain phenotype associated with *GPR56* heterozygous status in both humans and mice^12^,^13^,^19^,^20^. Furthermore, we observed the same number of EGFP^+^ OLs in the CC of *Gpr56*^*+/+*^ and *Gpr56*^+/−^ mice at P28 (Supplementary Fig. 2), confirming that loss of one allele of *Gpr56* causes no OL phenotype. Thus, we used *Gpr56*^+/−^ mice as littermate controls whenever possible to conserve animals. Staining for FluoroMyelin, a stain for compact myelin, was significantly decreased in the CC of *Gpr56*^−/−^ mice, compared with controls (Fig. 1a,b), suggestive of hypomyelination. Similarly, immunohistochemistry (IHC) and western blot analysis of myelin basic protein (MBP) and proteolipid protein (PLP), two markers for mature OLs and CNS myelin, showed significant reductions of the two proteins in *Gpr56*^−/−^ mice compared with controls (Fig. 1c–e and Supplementary Fig. 3).

### *Gpr56* mutants possess fewer myelinated axons

**Figure 2:**
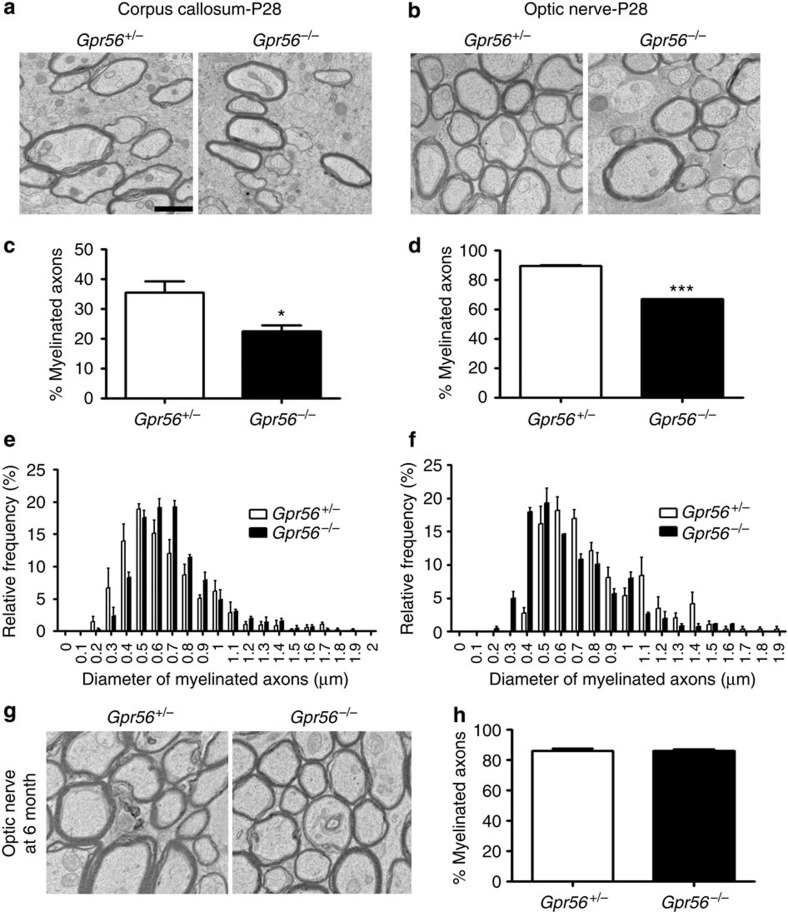
Loss of GPR56 results in fewer myelinated axons in the CC and optic nerves at P28. (**a**,**b**) Representative TEM images from P28 CC (**a**) and P28 optic nerves (**b**) of *Gpr56*^+/−^ (left) and *Gpr56*^−/−^ (right) mice. (**c**,**d**) Percentages of myelinated axons were quantified in the CC (**c**) and optic nerves (**d**) of *Gpr56*^−/−^ mice (*P=0.0264 (**c**); ***P=0.0004 (**d**); paired t-test, *n*=3 per genotype). (**e**,**f**) The distribution of myelinated axons with respect to the axon diameter was comparable in the CC (**e**) and optic nerve (**f**) between *Gpr56*^+/−^ and *Gpr56*^−/−^ mice (*P*=0.3185 (**e**); *P*=0.321 (**f**); paired *t*-test, n =3 per genotype). (**g**) Representative TEM images from the optic nerves at 6 months of *Gpr56*^+/−^ (left) and *Gpr56*^−/−^ (right) mice. (**h**) Percentage of myelinated axons was quantified (*P*=0.7680; paired *t*-test, *n*=3 per genotype). Error bars are means ±s.e.m. Scale bar, 1 μm.

To further assess CNS myelination phenotype in *Gpr56*-knockout mice, we performed transmission electron microscopy (TEM) analysis on cross-sections of the CC and the optic nerves of P28 *Gpr56*^+/−^ and *Gpr56*^−/−^ mice (Fig. 2a,b). Strikingly, there were significantly fewer myelinated axons in both the CC and optic nerves of *Gpr56*^−/−^ mice compared with the controls (Fig. 2c,d). To evaluate whether axons of a certain caliber were more severely affected in the absence of GPR56, we quantified the relative frequency of myelinated axons with respect to their corresponding diameters. We detected no statistical difference between the two groups (Fig. 2e,f), although we observed a shift towards a higher caliber of axons being myelinated in the CC of *Gpr56*^−/−^ mutants (Fig. 2e). Despite the significant reduction in the percentage of myelinated axons in *Gpr56*^−/−^ mutants, g-ratio analysis revealed no difference in myelin sheath thickness between the two groups (Supplementary Fig. 4a,b). We also observed no difference in axon diameter and the total number of axons (myelinated and unmyelinated) in the CC and optic nerves between the two groups of animals (Supplementary Fig. 4c–f). Interestingly, we observed normal levels of myelination at 6 months of age in the optic nerve (Fig. 2g,h), indicating the myelination defect was gradually corrected by ongoing OL production. Together, these data demonstrate that *Gpr56* mutants are hypomyelinated at early stages and that this phenotype is not due to gross axon defects.

### GPR56 is expressed in the OL lineage

**Figure 3:**
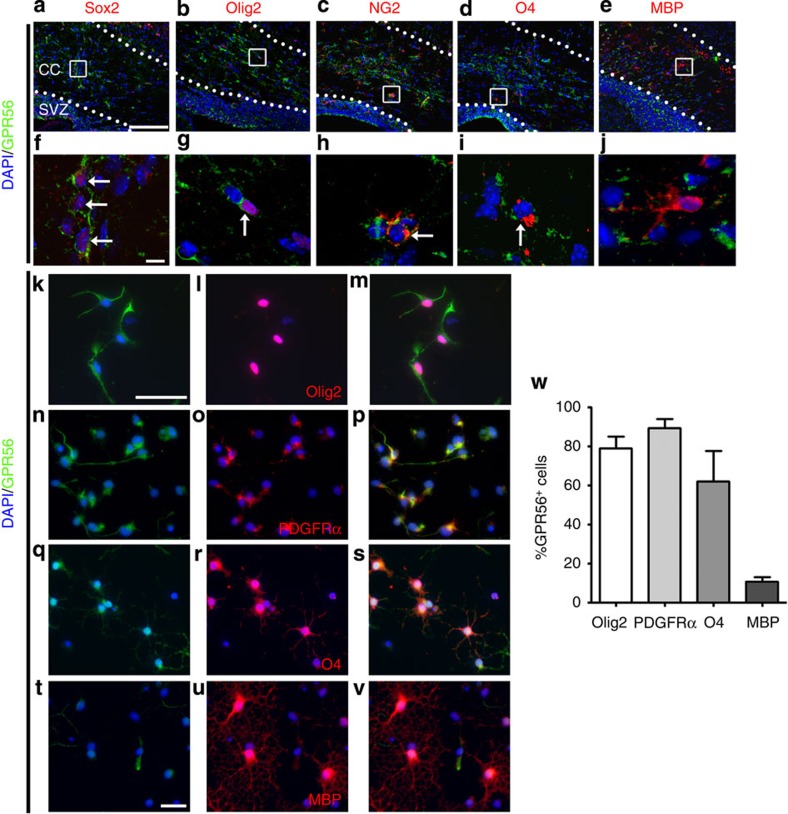
GPR56 is expressed in the OL lineage. (**a**–**e**) Double IHC for GPR56 (green) and Sox2, Olig2, NG2 and O4 (red) on wt P5 brains as well as MBP (red) on wt P10 brains. (**f**–**j**) Higher magnification of the boxed area in **a**–**e**. Scale bar, **a**–**e**, 100 μm; **f**–**j**, 10 μm. (**k**–**v**) Double immunocytochemistry of GPR56 (green) and various markers (red) on cultured primary OPCs and OLs that were either cultured for 2–4 days in proliferating media (**k**–**p**) or 3 days in differentiation media (**q**–**v**). Scale bar, 50 μm. (**w**) Bar graph depicts percentage of OL lineage cells expressing GPR56. CC, corpus callosum; SVZ, subventricular zone. Error bars are means ±s.e.m.

The observation that *Gpr56* mutation causes a reduction in the percentage of myelinated axons but not in the total number of axons suggests that GPR56 could regulate OL development. To test this hypothesis, we performed GPR56 expression profiling in the OL lineage by a series of both *in vivo* and *in vitro* immunostaining for GPR56 and markers of various stages of OL differentiation. Sox2 was used for a glial progenitor cell marker^25^, Olig2 for the OL lineage^26^,^27^,^28^, PDGFRα and NG2 for OPCs^29^, O4 antigen for immature OLs^30^ and MBP^31^ for mature myelinating OLs. In the CC of wild-type (wt) postnatal day (P) 5 mouse, GPR56 was detected in Sox2^+^, Olig2^+^, NG2^+^ and O4^+^ cells (Fig. 3a–d,f–i). By P10, the cells had matured into myelinating MBP^+^ OLs, GPR56 could no longer be detected (Fig. 3e,j). To further verify this temporal expression profile and to perform quantitative evaluation of GPR56 expression at various stages of OL differentiation, we performed double immunostaining on wt OPCs, immature and mature OLs (Fig. 3k–v and Supplementary Fig. 5). GPR56 was detected in ~80% of Olig2^+^ and ~90% PDGFRα^+^ cells (Fig. 3k–p,w). The percentage of GPR56^+^ cells steadily decreased in O4^+^ immature and MBP^+^ mature OLs (Fig. 3q–w). Taken together, our results indicate that GPR56 is expressed in glial progenitors and most OPCs and that this expression is downregulated in mature myelinating OLs, consistent with recent RNA-sequencing transcriptome data^32^. These results further support the notion that GPR56 could regulate OL development. Furthermore, our data showed ~10% PDGFRα^+^ OPCs do not express GPR56, suggesting a heterogeneous nature of OPC population. This expression profile also explains the correction of myelination defects in mutants at 6 months of age (Fig. 2g,h).

### Loss of *Gpr56* results in fewer mature OLs in the CC

**Figure 4:**
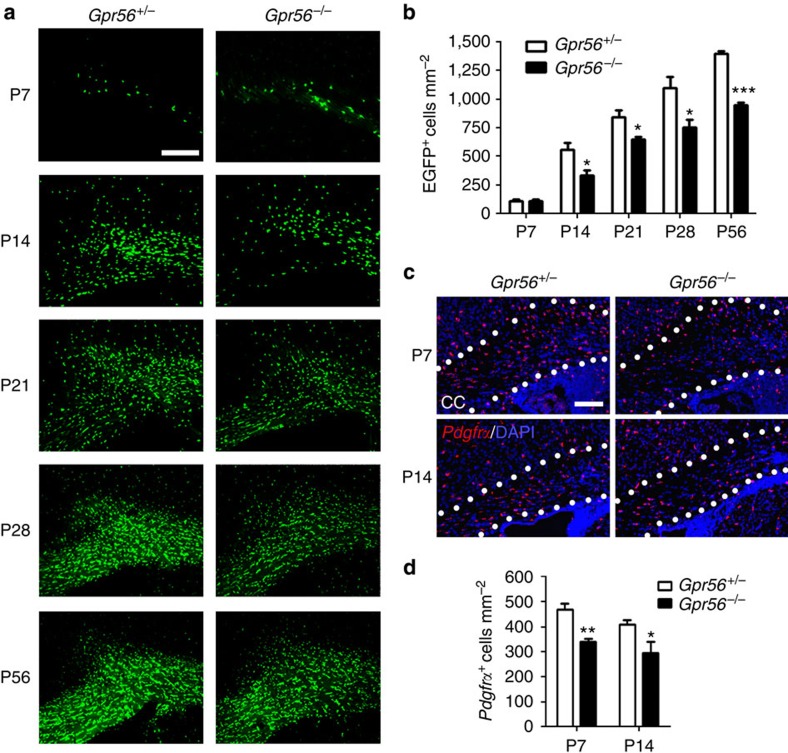
Loss of GPR56 results in fewer mature OLs and OPCs in the CC. (**a**) Representative images of EGFP^+^ (green) mature OLs in the CC of *Gpr56*^+/−^ (left panel) and *Gpr56*^−/−^ (right panel) mouse brains from P7 to P56. Scale bar, 200 μm. (**b**) Quantification of EGFP^+^ cells in the CC. P7 (*P*=0.9686), P14 (*P=0.0373), P21 (*P=0.0314), P28 (*P=0.0478) and P56 (***P<0.0001), unpaired *t*-test, *n*=3 per genotype. (**c**) Representative images of ISH of *Pdgfrα* (red). Scale bar, 100 μm. (**d**) Quantification of *Pdgfrα*^*+*^ cells in the CC. P7 (**P=0.0033), P14 (*P=0.0327), unpaired *t*-test, *n*=4 per genotype. Error bars are means ±s.e.m.

To test the hypothesis that GPR56 regulates OL development, we performed quantitative analysis of mature OLs in *Gpr56*-knockouts. We crossed *Plp:eGFP* transgenic reporter mice^33^ with *Gpr56*^−/−^ mice to generate *Plp:eGFP* /*Gpr56*^+/−^. Subsequent crossing with the F1 mice generated *Plp:eGFP* /*Gpr56*^+/+^, *Plp:eGFP*/*Gpr56*^+/−^ and *Plp:eGFP* /*Gpr56*^−/−^ mice. In these mice, enhanced green fluorescent protein, driven by the *Plp* promoter, mostly labels mature OLs^34^. At P7, we did not observe any difference between the number of EGFP^+^ OLs in the CC of mice of *Plp:eGFP* /*Gpr56*^+/−^ and *Plp:eGFP* /*Gpr56*^−/−^. In contrast, we observed significantly fewer EGFP^+^ OLs in the CC of *Gpr56*^−/−^ mice, compared with the controls, starting at P14 and continuing to P56 (Fig. 4a,b). Next, we quantified the total number of OPCs by performing *Pdgfrα in situ* hybridization (ISH) on P7 and P14 *Gpr56*^−/−^ mice and their littermate controls. We observed a significant reduction in the number of *Pdgfrα*^+^ cells in the CC of *Gpr56*^−/−^ mice compared with the controls at both developmental stages analysed (Fig. 4c,d). To evaluate the status of immature OLs in the CC of *Gpr56*-knockout mice, we measured levels of 2′,3′-cyclic nucleotide 3′-phosphodihydrolase (CNP), a marker for immature OLs^1^,^26^. We observed reduced CNP protein levels in the CC of *Gpr56*^−/−^ mice, compared with their littermate controls (Supplementary Fig. 6), indicative of fewer CNP^+^ immature OLs. Together, these data demonstrate that GPR56 is required for the proper development of OLs.

### GPR56 is required for OPC proliferation

**Figure 5:**
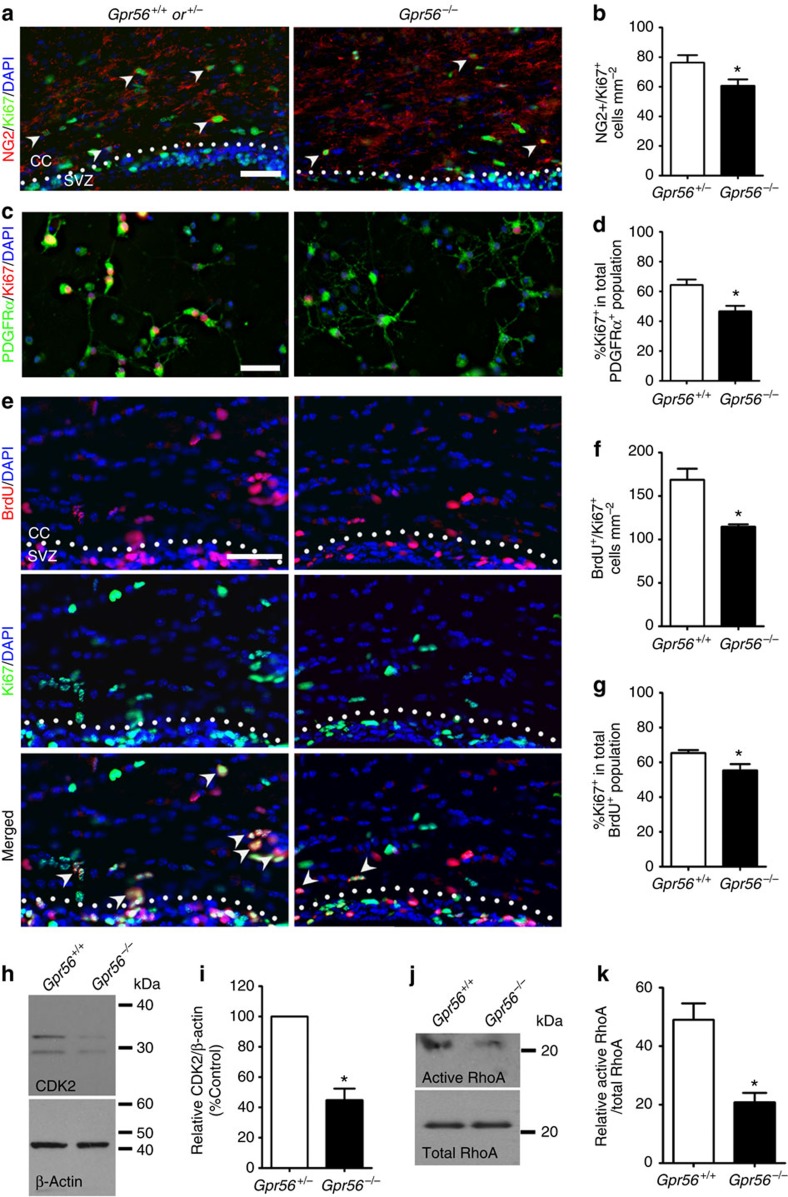
Loss of GPR56 leads to fewer proliferating OPCs. (**a**) Representative images of NG2 (red) and Ki67 (green) double IHC in the CC of *Gpr56*^+/−^ and *Gpr56*^−/−^ P14 mice. Arrowheads indicate double-positive cells. (**b**) Quantification of NG2 and Ki67 dual-positive cells. The asterisks represent significance based on unpaired *t*-test. *P*=0.0382; *n*=6 per genotype. (**c**) Representative images of PDGFRα (green) and Ki67 (red) double immunostaining on OPCs after cultured for 4 days in proliferation media. (**d**) Quantification of PDGFRα and Ki67 dual-positive OPCs. The asterisks represent significance based on paired *t*-test. *P*=0.0156; *n*=3 per genotype. (**e**) Representative images of BrdU (red) and Ki67 (green) double staining on P14 *Gpr56*^*+/+*^ and *Gpr56*^−/−^ brains that were pulsed with BrdU 24 h before. Arrowheads indicate double-positive cells. (**f**) The number of BrdU and Ki67 double-positive cells was quantified in the CC of *Gpr56*^−/−^ mice compared with controls. The asterisks represent significance based on unpaired *t*-test. *P*=0.0153; *n*=3 per genotype. (**g**) The percentage of Ki67^+^ in the total BrdU^+^ cell population was quantified in the CC of *Gpr56*^−/−^ mice compared with the *Gpr56*^*+/+*^ controls. The asterisks represent significance based on unpaired *t*-test. *P*=0.0289; *n*=4 per genotype. (**h**) Western blot depicting CDK2 protein level in actually isolated OPCs from *Gpr56*^*+/+*^ and *Gpr56*^−/−^ P6 mice. (**i**) The relative CDK2 protein levels were shown. The asterisks represent significance based on paired *t*-test. *P*=0.0179; *n*=3 per genotype. (**j**) Western blot of active RhoA (top panel) and total RhoA (bottom panel) in the optic nerves of *Gpr56*^*+/+*^ and *Gpr56*^−/−^ mice. (**k**) The relative level of active RhoA to total RhoA was presented. The asterisks represent significance based on unpaired *t*-test. *P*=0.0122; *n*=3 per genotype. CC, corpus callosum; SVZ, subventricular zone. Scale bar, 50 μm. Error bars are means ± s.e.m.

GPR56 was recently shown to regulate neural stem cell proliferation in the developing neocortex^21^. To test whether the reduced number of OPCs and mature OLs observed in *Gpr56* knockouts is due to decreased OPC proliferation, we performed double immunostaining of NG2 and Ki67, markers for OPCs and proliferating cells, respectively, on postnatal brains of *Gpr56*^−/−^ and their littermate controls. We chose to examine P14 brains based on our observation that fewer mature OLs were first observed at this stage (Fig. 4). A significantly reduced number of dual-positive cells was detected in *Gpr56*^−/−^ brains, compared with the controls (Fig. 5a,b), suggesting that GPR56 has a role in regulating OPC proliferation, either directly or indirectly.

Next, we carried out cell proliferation assays *in vitro* using OPCs isolated from P5 brains of *Gpr56*^−/−^ mice and their littermate controls by immunopanning^35^,^36^,^37^. After culturing for 4 days in the presence of PDGF-AA, a major OPC mitogen, we found that the percentage of dividing OPCs, represented by Ki67^+^ in total PDGFRα^+^ population, was significantly reduced in OPCs derived from *Gpr56*^−/−^ mouse brains compared with controls (Fig. 5c,d).

In light of the expression pattern of GPR56 in OL development and the reduced OPC proliferation in the absence of GPR56, we hypothesized that GPR56 is required for OPCs to remain in cell cycle. To test this hypothesis, we performed cell cycle exit assay by pulsing *Gpr56*^−/−^ pups and their wt littermate controls with BrdU on P13, 24 h before brain harvesting, followed by double IHC of BrdU and Ki67. BrdU and Ki67 dual-positive cells represent those cells that remain in the cell cycle, whereas BrdU^+^;Ki67^−^ cells represent those that have already exited the cell cycle at the time of analysis. We found significantly fewer BrdU^+^;Ki67^+^ double-positive cells (Fig. 5e,f) as well as a lower percentage of Ki67^+^ cells relative to the total BrdU^+^ population (Fig. 5g) in the CC of *Gpr56*^−/−^ mice in contrast to their littermate controls. Cyclin-dependent kinase 2 (CDK2) controls OPC cell cycle progression^38^,^39^. To further demonstrate that GPR56 keeps OPCs in cell cycle, we performed western blot analysis of CDK2 in acutely isolated OPCs from P6 *Gpr56*^−/−^ mice and their littermate controls by immunopanning. We observed a significant reduction of CDK2 protein in the OPCs isolated from *Gpr56*^−/−^ mice compared with their littermate controls (Fig. 5h,i), supporting that OPCs prematurely exit the cell cycle in the absence of GPR56.

### GPR56 promotes OPC proliferation via the RhoA pathway

Two signalling pathways have been reported to be downstream of GPR56 activation, depending on the cell type. In neural progenitor cells, GPR56 signals via the RhoA pathway^40^, whereas it activates protein kinase Cα (PKCα) in melanoma cells^41^. To investigate the signalling mechanism of GPR56 in the developing white matter, we performed active RhoA pull down assays and PKCα western blot analyses, using P7 optic nerves of *Gpr56*^−/−^ mice and their littermate controls. Whereas the level of PKCα was not affected by deleting *Gpr56* (Supplementary Fig. 7a,b), we observed significantly lower levels of active RhoA in the optic nerves of *Gpr56*^−/−^ mice compared with their littermate controls (Fig. 5j,k). These data suggest that GPR56 signals through RhoA to regulate OPC proliferation.

### GPR56 has no effect on OPC survival and maturation

**Figure 6:**
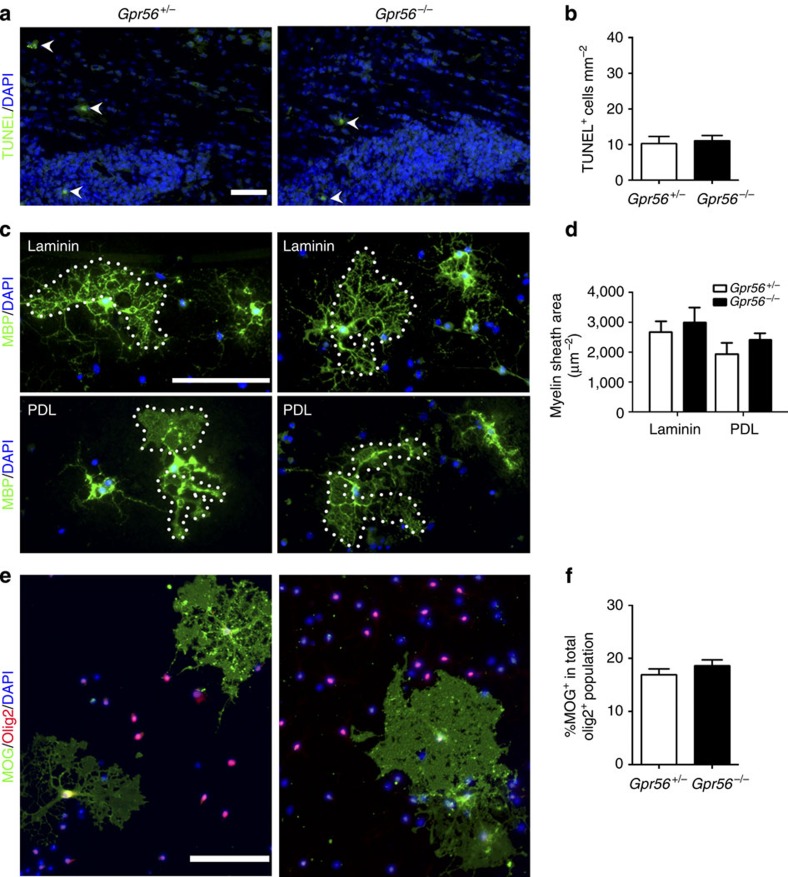
Loss of GPR56 has no effect on OPC survival, OL process elaboration and maturation. (**a**) Representative images of TUNEL^+^ cells (green; arrowheads) in the SVZ and CC of *Gpr56*^+/−^ and *Gpr56*^−/−^ mice at P14. Scale bar, 50 μm. (**b**) Quantification of TUNEL^+^ cells. *P*=0.7573; unpaired *t*-test, *n*=6 per genotype. (**c**) Representative images of MBP^+^ (green) OLs cultured for 7 days on either laminin (upper panel) or poly-D-lysine (PDL, lower panel) in differentiation media. Scale bar, 100 μm. (**d**) Quantification of myelin sheath area of OLs cultured on laminin (*P*=0.2043) or PDL (*P*=0.3027) (paired *t*-test, *n*=3 per genotype). (**e**) Representative images of MOG (green) and Olig2 (red) double labelling on OLs derived from *Gpr56*^+/−^ and *Gpr56*^−/−^ mice cultured for 7 days in differentiation medium. Scale bar, 100 μm. (**f**) Percentage of differentiated OLs (MOG^+^) in total OLs (Olig2^+^) was quantified. *P*=0.5044; paired *t*-test, *n*=3 per genotype. Error bars are means ± s.e.m.

Another potential cause for reduced mature OLs in *Gpr56* mutants is increased OPC cell death in the absence of *Gpr56*. Cell death assays revealed comparable numbers of TUNEL-positive apoptotic cells in the subventricular zone and corpus callosum (CC) in both control and *Gpr56*^−/−^ brains at P14 (Fig. 6a,b). Furthermore, judging by the similar distribution pattern of *Pdgfrα*^+^ cells at P14 (Fig. 4c) as well as EGFP^+^ cells from P7 to P56 (Fig. 4a), in the CC of *Gpr56*^+/−^ and *Gpr56*^−/−^ mice, GPR56 likely has no influence on OPC migration.

In addition, we suspected that GPR56 does not affect the elaboration of branched processes of OLs based on the fact that GPR56 is downregulated in terminally differentiated MBP^+^ OLs (Fig. 3) and that deleting *Gpr56* has no effect on the myelin thickness (Supplementary Fig. 4a,b). To confirm this notion, we cultured OPCs isolated from *Gpr56*^−/−^ and their littermate controls on either poly-D-lysine (PDL) or laminin-coated coverslips for 7 days in the presence of thyroid hormone to induce terminal differentiation. Indeed, we did not observe any difference in the myelin sheath area between *Gpr56*^+/−^ and *Gpr56*^−/−^ OLs cultured on laminin or PDL (Fig. 6c,d). Moreover, deleting *Gpr56* has no effect on the ability of OPCs to terminally differentiate (Fig. 6e,f).

### GPR56 functions autonomously in the OL lineage

**Figure 7:**
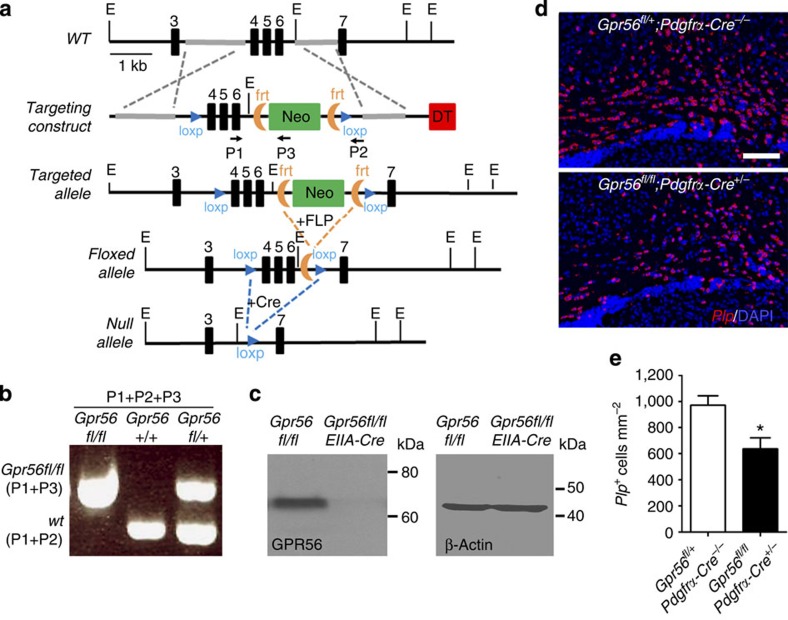
OPC-specific deletion of *Gpr56* leads to fewer mature oligodendrocytes. (**a**) Schematic drawing of targeting strategy. Exons 4, 5 and 6 were flanked with two loxP sites. (**b**) PCR genotyping revealed *Gpr56*^*fl/fl*^, *Gpr56*^*+/+*^ and *Gpr56*^*fl/+*^ alleles. (**c**) Western blot analysis shows absence of GPR56 protein in *Gpr56*^*fl/fl*^*;EIIA-Cre*^+/−^. (**d**) ISH of *Plp* (red) in the CC of P21 *Gpr56*^*fl/fl*^*;Pdgfrα-Cre*^+/−^ mice (lower panel) and *Gpr56*^*fl/+*^*;Pdgfrα-Cre*^−/−^ controls (upper panel) that received tamoxifen for five consecutive days during P10-P14. Scale bar, 100μm. (**e**) Quantification of *Plp*^*+*^ mature OLs. The asterisks represent significance based on unpaired *t*-test. *P*=0.0177; unpaired *t*-test, *n*=4 per genotype. Error bars are means ±s.e.m.

To define the cell autonomy of GPR56 during OL development, we generated a new targeted allele of *Gpr56* containing *loxP* sites flanking exons 4–6, hereafter referred to as the floxed (*fl*) allele of *Gpr56* (Fig. 7a,b). On crossing with tissue-specific *Cre* transgenic mice, exons 4–6 are deleted, causing a frameshift leading to a deletion of all splicing variants of *Gpr56*. We first crossed *Gpr56*^*fl/fl*^ mice with *EIIA-Cre* mice, a universal Cre-line^42^, to create a constitutive knockout mouse line. Western blot analysis failed to detect any GPR56 protein in the brains of *Gpr56*^*fl/fl*^*;EIIA-cre*^+/−^ mice (Fig. 7c), confirming the efficacy of our targeting strategy. We began our analysis using *Pdgfrα-CreERT* transgenic mice^43^,^44^ to excise *Gpr56* in OPCs by daily administration of tamoxifen from P10 to P14. We chose P10-14 based on the observations that oligodendrogenesis starts perinatally and peaks at P14 (refs 45, 46, 47, 48). We performed *Plp* ISH on P21 CC of *Gpr56*^*fl/fl*^*;Pdgfrα-cre*^+/−^ and their littermate controls (Fig. 7d). A significantly fewer number of *Plp*^+^ mature OLs was found in the CC of *Gpr56*^*fl/fl*^*;Pdgfrα-cre*^+/−^, compared with the controls (Fig. 7e), demonstrating that GPR56 regulates OL development in a cell-autonomous manner.

## Discussion

Whereas a clear role has been established for GPR56 in cerebral cortical development^12^,^19^,^40^,^49^,^50^, nothing was known about its function in CNS myelination. We demonstrate here that GPR56 is a novel regulator of OL development. Consistent with a previous report that GPR56 promotes neural stem cell proliferation in the developing neocortex^21^, we show that developing OPCs require GPR56 to remain in a proliferating state by demonstrating that (1) GRP56 is expressed robustly in Sox2^+^, NG2^+^ and PDGFRα^+^ cells, diminished in O4^+^ cells and strongly downregulated in MBP^+^ OLs. (2) There are significantly fewer NG2^+^;Ki67^+^ double-positive cells in the CC of *Gpr56* knockouts. (3) Fewer PDGFRα^+^ OPCs derived from *Gpr56* knockouts proliferate following 4 days of culture *in vitro*. (4) Loss of GPR56 causes OPCs to exit cell cycle prematurely. (5) Conditionally ablating *Gpr56* in OPCs results in a significantly reduced number of mature OLs. The fact that deleting *Gpr56* has no effect on axon diameter or the total number of axons further supports that GPR56 has an autonomous function in OPC development.

ECM and ECM receptor loss-of-function studies in mice have demonstrated that cell–matrix interactions are important for gliogenesis and myelination^3^. For example, many studies have shown that laminin–integrin interactions regulate OL process dynamics^3^ and *dy/dy* mutants (*α2 laminin* hypomorphs) have regional defects in CNS myelination as well as delayed OL maturation^4^,^5^. These published studies are consistent with the phenotypes that we observed in *Gpr56*^−/−^ mutants. Moreover, aGPCRs often bind ECM proteins and we hypothesize that GPR56–ECM interactions regulate OL development. It is unclear which ECM ligand activates GPR56 in the developing white matter. Collagen III is the ligand of GPR56 in the developing cerebral cortex. However, collagen III is mainly expressed in the meninges and blood vessels^40^, making it an unlikely ligand of GPR56 during OPC development.

GPR56 has a very long and poorly characterized N-terminal fragment, allowing for the possibility of multiple binding partners. Indeed, GPR56 also binds to tissue transglutaminase (TG2) in melanoma cells^51^. TG2 is an inducible transamidating acyltransferase that has several distinct biochemical functions^52^,^53^,^54^. Interestingly, the enzymatic activity of TG2 is low in the early embryonic mouse brain but increases through development, peaks on the day of birth (P0) and is maintained at high levels up to P56 (ref. 55), consistent with a potential role in OL development and myelination. TG2 activity has recently been implicated in CNS remyelination, as *Tg2*^−/−^ mice remyelinate more slowly than wt animals in cuprizone models^56^. Moreover, OPC differentiation is inhibited *in vitro* on addition of TG2 inhibitors^56^. Therefore, it is possible that TG2 functions as the ligand of GPR56 during white matter development. Future studies are needed to test this hypothesis.

Further elucidation of GPR56 signalling in OPCs depends on the identification of its ligand. Gα_12/13_ and RhoA are the downstream effectors of GPR56 in neural progenitor cells and cultured cell lines^40^,^57^,^58^. On the basis of the fact that inactivation of RhoA is required for the terminal differentiation of OLs^59^,^60^,^61^,^62^ and that GPR56 ceases to be expressed in mature myelinating OLs, RhoA could signal downstream of GPR56 in the developing OPCs. Indeed, we detected significantly reduced level of active RhoA in the optic nerves of *Gpr56*^−/−^ mice compared with their littermate controls.

**Figure 8:**
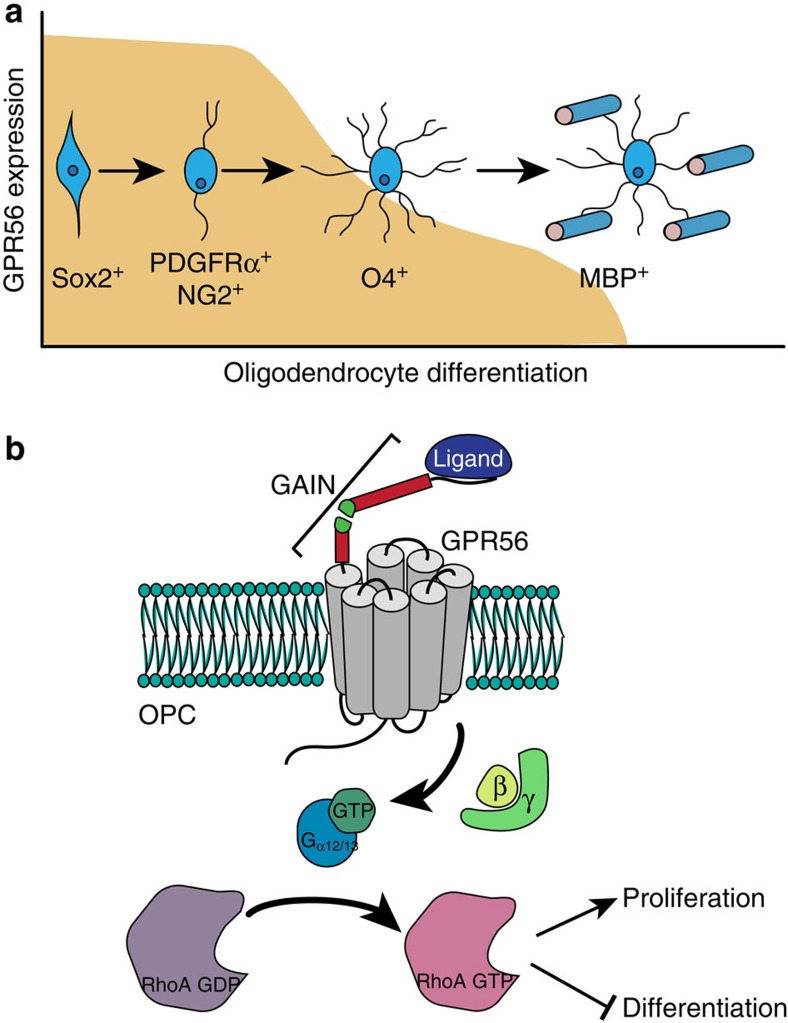
GPR56 keeps OPCs in a proliferative state. (**a**) GPR56 is mainly expressed in NG2^+^/PDGFRα^+^ OPCs. Its expression is downregulated beginning at the immature O4^+^ OL stage. The shaded area depicts the developmental stages where GPR56 expression is detected. (**b**) Model of GPR56 function in OPCs. GPR56, on binding to its unknown ligand, promotes OPC proliferation by activating RhoA.

In summary, we reveal a novel GPCR that regulates proper levels of CNS myelination by autonomously promoting OPC proliferation via the RhoA pathway (Fig. 8). Identification and characterization of signalling molecules involved in OL development may provide potential therapeutic targets for enhancing remyelination. Given the fact that GPCRs are the major targets for drug discovery^63^, the present study presents a potential new target for therapeutics to promote myelin repair in individuals afflicted with demyelination or dysmyelination.

## Methods

### Mice

All animals were treated according to the guidelines of the Animal Care and Use Committee at Boston Children’s Hospital. The *Gpr56*-knockout mice were obtained from Genentech/Lexicon Genetics. The mutant mice were originally created in a 129/BL6 background, but were derived into the FvB strain and bred into BALB/c strain resulting in a mixed genetic background of the mutant mice of 129/BL6/FvB/BALB/c^19^. Genotyping was performed by PCR using the following primers: A (5′- CGAGAAGACTTCCGCTTCTG -3′), B (5′- AAAGTAGCTAAGATGCTCTCC -3′) and Neo (5′- GCAGCGCATCGCCTTCTATC -3′). *Plp:eGFP* transgenic mice^33^ were bred into the *Gpr56* line to create *Plp:eGFP/Gpr56*^*+/+*^*, Plp:eGFP/Gpr56*^+/−^ and *Plp:eGFP*/*Gpr56*^−/−^ mice.

*Gpr56*^*fl/+*^ mice were generated at the Mouse Gene Manipulation Core at Boston Children’s Hospital. The targeting vector (Fig. 7a) was introduced into *C57BL/6* ES cells to generate targeted ES clones. Chimeric mice derived from targeted ES cell clone were crossed with *C57BL/6* mice to obtain *Gpr56*^*fl/+*^ mice. We used the following primers to detect the presence of the floxed allele: primer 1: 5′- tggtagctaacctactccaggagc -3′, primer 2: 5′- ggtgactttggtgttctgcacgac -3′ and primer 3: 5′- cacgagactagtgagacgtgctac -3′.

*Pdgfrα-Cre/ERT* mice in a C57BL/6 background were purchased from Jackson Laboratories (Cat# 018280) and were crossed with *Gpr56*^*fl/fl*^ mice to generate *Gpr56*^*fl/fl*^;*Pdgfrα-Cre*^+/−^ mice and their littermate controls. *EIIA-Cre* mice in a C57BL/6 background were purchased from Jackson Laboratories (Cat# 003724) and were crossed with *Gpr56*^*fl/fl*^ mice to generate *Gpr56*^*fl/fl*^*;EIIA-Cre*^+/−^ mice and controls. Only male mice were used for time points of P28 and later to avoid cyclic oestrogen effect on myelination, whereas both male and female mice were used for earlier time points.

### Antibodies

The following primary antibodies were used for IHC or western blot analyses: mouse anti-GPR56 (H11) (1:200)^40^ and rabbit anti-GPR56 (199) (1:200)^19^, rabbit anti-MBP (Millipore; Cat #AB980, 1:200), rat anti-MBP (Abcam, Cat# ab7349), mouse anti-O4 (Millipore; Cat #MAB345, 1:400), rabbit anti-NG2 (Millipore; Cat #AB5320, 1:200), goat anti-Sox2 (Santa Cruz; Cat #sc-17320, 1:400), rabbit anti-Olig2 (kind gift from Charles Stiles, 1:10,000), rat anti-PDGFRα (BD Bioscience; Cat #558774, 1:500), rabbit anti-PDGFRα (Cell Signaling Technologies; Cat #3164S, 1:500) and rat anti-Ki67 (Affymetrix eBioscience; Cat #14-5698-80, 1:100), rat anti-BrdU (Accurate Chemical and Scientific Corporation; Cat #OBT0030S, 1:100), rabbit anit-PLP (Abcam, Cat #ab28486, 1:1,000), mouse anti-RhoA (Cytoskeleton, Cat# ARH03-A, 1:500), mouse anti-CDK2 (Santa Cruz; Cat #sc-6248, 1:1,000), mouse anti-β-actin (Sigma, Cat #A5044, 1:5,000) and mouse anti-Ki67 (BD Bioscience; Cat #550609, 1:100). Secondary antibodies were goat anti-mouse or anti-rat conjugated with either Alexa 488 (Life Technologies, 1:1,000) or Alexa 546 (Life Technologies, 1:1,000) and goat anti-rabbit conjugated with Alexa 546 or 555 (Life Technologies, 1:1,000), goat anti mouse or rabbit IgG-HRP (Sigma, Cat# A4416 or A6154, 1:3,000).

### Histology analyses

Mouse brains were harvested after perfusion, fixed with 4% PFA, cryoprotected with 30% sucrose and embedded in OCT. IHC was carried out as previous described^64^. In brief, after antigen retrieval in Retrievagen A Solution (BD Pharmingen), brain sections were washed with PBS, blocked with 10% goat serum, 1% bovine serum albumin (BSA) and 0.1% Triton X-100 in PBS for 1 h at room temperature before incubating with the primary antibody overnight at 4 °C. Primary antibodies were visualized by incubating the sections with the appropriate fluorophore-conjugated secondary antibody for 1 h at room temperature followed by staining of the nuclei with Hoechst 33342 (1:2,000, Life Technologies).

TUNEL assays (Millipore) were performed on 12 μm-thick brain sections of P14 mouse brains post-fixed for 35 min in ethanol and acetic acid, according to the manufacturer’s protocol.

OPCs and OLs were fixed in 2% PFA, followed by subsequent double immunostaining as previously described^64^. All images were captured using a confocal LSM 510 NLO system or a Nikon Eclipse Ti inverted microscope (Nikon). Representative photographs were obtained with the same exposure setting for control and mutant.

*In situ* hybridization was performed on 12 μm-thick brain sections as previously described^65^,^66^ . Probes targeting *Plp* (Addgene, Cat #22651) and *Pdgfrα* (kind gift from Charles Stiles) were generated by digesting the plasmids with EcoRI and HindIII, respectively. DIG-labelled RNA probes were generated using Sp6 and T7 polymerase *in vitro* transcription (Roche Applied Science; DIG RNA labelling kit) as per manufacturer’s instructions. Hybridization occurred at 68 °C and washes at 65 °C. To detect the DIG-labelled probes, the TSA-Plus Cyanine 3 labelling system (Perkin Elmer) was used according to the manufacturer’s instructions.

FluoroMyelin Fluorescent Myelin Stains (Life Technologies) were performed on 12 μm-thick brain sections of P14 and P28 *Gpr56*^−/−^ pups and *Gpr56*^+/−^ littermate controls according to the manufacturer’s protocol. Percentage area myelinated was quantified by outlining the CC and determining the number of pixels brighter than +10 a.u. (0–255) above background per total number of pixels as previously described^65^.

### Tamoxifen treatment and cell cycle exit assay

A solution of 10 mg ml^−1^ tamoxifen (Sigma; T5648) was prepared in corn oil. Male and female pups were injected intraperitoneally once daily for five consecutive days at 50 mg kg^−1^ body weight from P10 to P14. Mouse brains were harvested on P21, 7 days after the last tamoxifen injection.

Cell cycle exit assays were performed as previously described^67^. In brief, proliferating cells were labelled with BrdU (50 mg kg^−1^) by intraperitoneal injection of male and female P13 *Gpr56*^−/−^ pups and their littermate controls. After 24 h, mouse brains were harvested after perfusion, fixed with 4% PFA, cryoprotected with 30% sucrose and embedded in OCT. Brain sections were processed for IHC with anti-BrdU and anti-Ki67 antibodies.

### Western blot and GTP-Rho pull-down assay

The CCs were dissected under a Leica stereo microscope (MZ 6; Leica Pte), followed by washes in PBS and lysis in ice-cold RIPA buffer (1% Nonidet P-40, 50 mM Tris pH 7.6, 120 mM NaCl, 1 mM EDTA) containing protease inhibitor cocktail set 1 (Calbiochem). The lysates were cleared of insoluble materials by centrifugation at 16,000 *g* for 10 min at 4 °C. Protein concentration was determined by a Bio-Rad protein assay method (Bio-Rad) according to the manufacturer’s protocol, and equal amount of proteins were used for SDS–PAGE and western blot analysis. The GTP-Rho pull-down assay was performed as previously described^40^, using mouse optic nerves of male and female P7 *Gpr56*^−/−^ pups and their littermate controls. In brief, P7 mouse optic nerves were pooled according to their genotype into heterozygous and knockout groups. Tissues were grinded as a powder on liquid nitrogen and lysed in 300 μl of ice cold RIPA buffer containing protease inhibitors with a cell disruptor for 10 min and homogenization with syringe needle 26 G. An equal amount of total protein was incubated with 60 μg GST-RBD beads (Cytoskeleton) at 4 °C for 90 min. The beads were washed twice with lysis buffer and once with TBS buffer. Bound Rho proteins were eluted by Laemmli sample buffer and detected by western blotting using a mouse monoclonal anti-RhoA antibody (Cytoskeleton).

### Transmission electron microscopy

Postnatal male brains were fixed by immersion in mixture of 2.5% glutaraldehyde and 2% paraformaldehyde in 0.1 M sodium cacodylate buffer, pH 7.4. After overnight fixation, tissues were postfixed in 1% osmium tetroxide, then dehydrated and embedded in Epon-Araldite. Ultrathin sections were cut and stained with uranyl acetate and lead citrate. The samples were observed and photographed using the transmission electron microscope (Tecnai G^2^ Spirit BioTWIN) at the Harvard Medical School EM Facility. Optic nerves were processed and imaged as described^9^. The photographs were analysed using Image J Software (http://rsb.info.nih.gov/ij/) to calculate g-ratio and axon diameter. G-ratio was calculated as previously described^68^.

### OPC cultures

McCarthy-deVellis cultures were used for terminal differentiation experiments (Fig. 6e,f). OPCs were isolated from the forebrains of male and female P1 *Gpr56*^−/−^ pups and their littermate controls after genotyping, as previously described^69^. In brief, dissociated cells from the forebrains of P1 pups were cultured for 10 days at 37 °C and 8.5% CO_2_ before shaking at 45 r.p.m. and 37 °C for 1 h to remove microglia cells. Media was replaced with fresh DMEM/FBS, and the culture was again shaken at 250 r.p.m. and 37 °C for 18–20 h to harvest OPCs, followed by incubating on 10 cm Petri dish for 60 min at 37 °C to remove contaminating astrocytes and microglia. Purified OPCs were thoroughly dissociated, plated onto poly-D-lysine-coated coverslips and cultured for 7 days in thyroid hormone containing differentiation medium for terminal differentiation.

O4 panning cultures were performed for proliferation and process elaboration assays. OPCs were isolated from male and female P5-6 *Gpr56*^*+/+*^ or *Gpr56*^−/−^ mouse forebrains, as previously described^36^,^37^. Immunopanning was carried out using mouse anti-Thy1.2 (Serotec, Cat #MCA02R) and mouse anti-GalC (Millipore, Cat #MAB342) for negative selection, followed by mouse anti-O4 AB (O4 hybridoma supernatant) for positive selection. OPCs were released from the O4 plate by trypsinization and resuspended in media as previously described^35^. Purified OPCs were plated on PDL- or laminin-coated coverslips and cultured in proliferating media containing PDGF-AA and NT-3 (PreproTech) for 4 days or in differentiating medium containing thyroid hormone and B27 for 7 days. Cells were then fixed with 2% PFA and stained for PDGFRα and Ki67 or for MBP. The area of the myelin sheath of oligodendrocytes was measured using NIS-Elements 3.1 Advanced Research (Nikon) software as previously described^59^ by outlining the myelin sheath area (as indicated in Fig. 6c).

### Statistical analysis

For all studies, images were scored blinded to genotype before quantifications. Data are represented as mean±s.e.m. GRAPHPAD Prism Software (GraphPad Software) was used to determine statistical significance between genotypes using unpaired *t*-tests or paired *t*-tests, two-tailed and unequal variance depending on animals either being paired before data collection or not. For *in vitro* culture, animals were paired before isolation of OPCs. Statistical significance between genotypes was determined using paired *t*-tests, two-tailed and unequal variance. Sample size was not pre-determined by statistical methods, but was based on similar studies in the field.

## Additional information

**How to cite this article**: Giera, S. *et al*. The adhesion G protein-coupled receptor GPR56 is a cell-autonomous regulator of oligodendrocyte development. *Nat. Commun.* 6:6121 doi: 10.1038/ncomms7121 (2015).

## Supplementary Material

Supplementary Information Supplementary Figures 1-7
