# Supplementary material for: The adhesion G protein-coupled receptor GPR56 is a cell-autonomous regulator of oligodendrocyte development
Source: Nat Commun. 2015 Jan 21;6:6121. doi: 10.1038/ncomms7121 (PMC4302951; doi:10.1038/ncomms7121)
Supplement: Supplementary Information — Supplementary Figures 1-7 [file ncomms7121-s1.pdf]

Supplementary Information

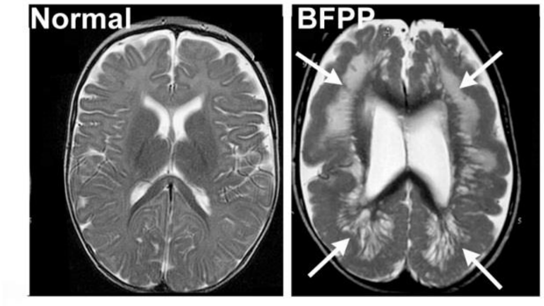

Supplementary Figure 1: CNS myelination defects are associated with *GPR56* mutation. Compared to the white matter in the normal brain (left), the BFPP brain (right) is characterized by reduced white matter volume as well as T2 signal changes (arrows) on the MRI, indicating defective myelination in the white matter tracts.

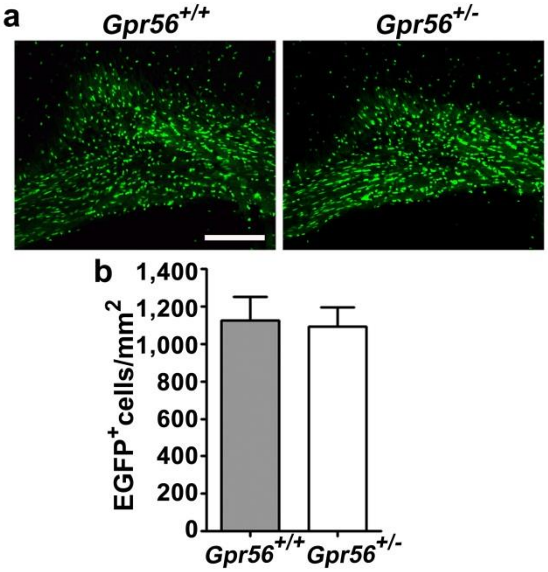

Supplementary Figure 2: The number EGFP$^+$ OLs is comparable between *Gpr56* wild type and heterozygous mice. (a) Representative images of EGFP$^+$ (green) cells in the CC of *Plp:eGFP/Gpr56*$^{+/+}$ and *Plp:eGFP/Gpr56*$^{+/-}$ mice at P28. Scale bar: 200 µm. (b) Quantification of EGFP$^+$ OLs. p=0.8598; unpaired t-test, n = 3 per genotype. Error bars are means ± s.e.m.

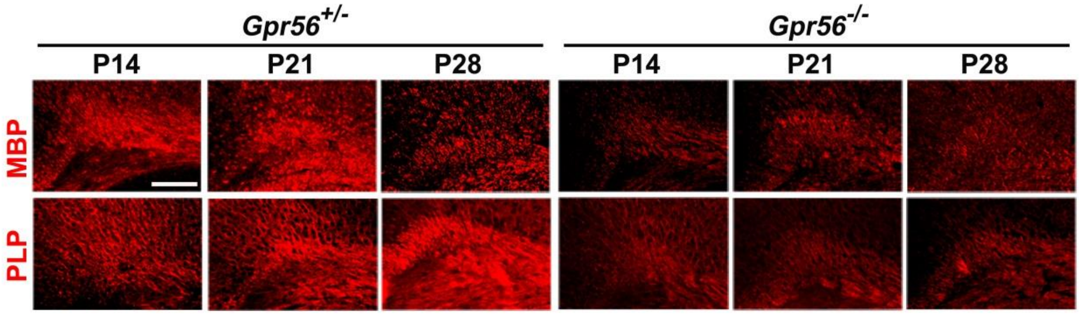

Supplementary Figure 3: Loss of GPR56 results in a reduced MBP and PLP protein level in the postnatal CC. IHC of MBP (red) and PLP (red) on the CC of *Gpr56*$^{+/-}$ and *Gpr56*$^{-/-}$ mice at P14, P21 and P28. Deleting *Gpr56* led to a reduced level of MBP and PLP in the CC of *Gpr56*$^{-/-}$ mice, compared to their heterozygous littermate controls. Scale bar: 200 µm.

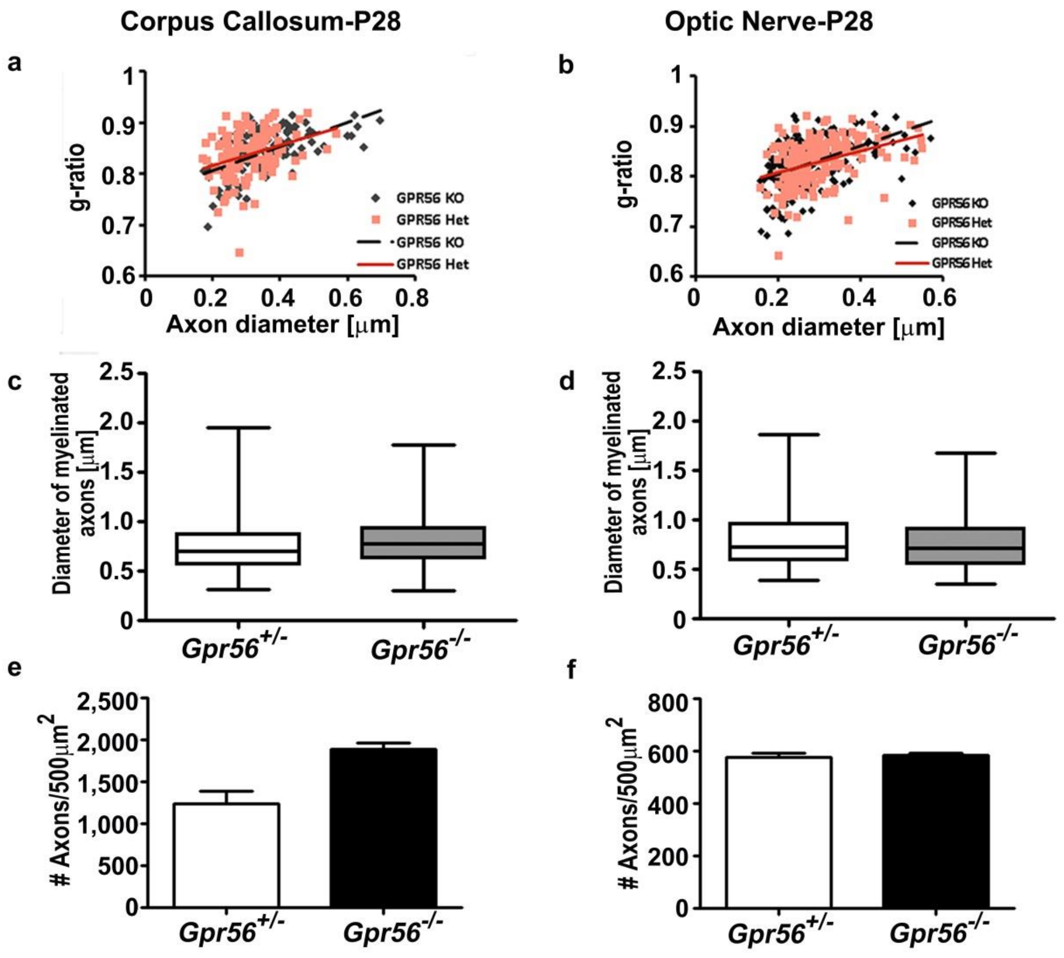

Supplementary Figure 4: Deleting *Gpr56* has no effect on myelin thickness and axon diameter in the CC and optic nerves. (a, b) Myelin thickness, presented as g-ratio in the CC (a) and optic nerves (b), was quantified. p=0.0961(c); p=0.9982 (d); paired t-test, n =3 per genotype. (c, d) Axon diameter in the CC (a) and optic nerve (b) was quantified. p=0.3119 (a); p=0.4272 (b); paired t-test, n =3 per genotype. (e, f) The number of total axons (myelinated and unmyelinated) in the CC (e) and optic nerve (f) was quantified. p=0.0942 (e), p=0.989 (f); paired t-test, n =3 per genotype. Error bars are means ± s.e.m.

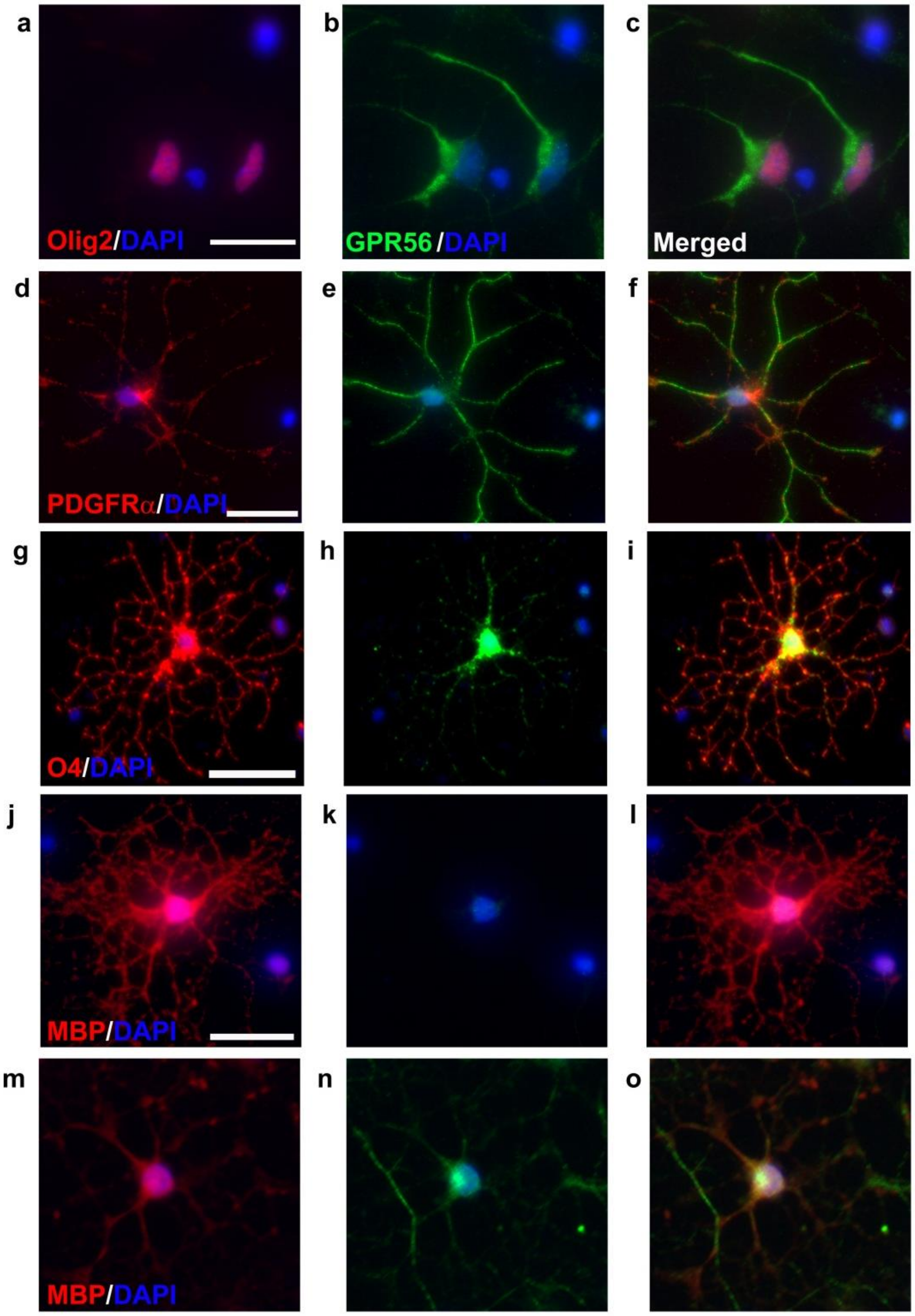

Supplementary Figure 5: The expression of GPR56 in cultured OPCs and OLs.

Immunocytochemistry of cultured OPCs and OLs that were either cultured for 2-4 days in proliferating media (a-i) or 3 days in differentiation media (j-o). GPR56 (green) is expressed in Olig2$^+$ (a-c, red), PDGFRα^+ (d-f, red) and O4$^+$ (g-i, red). Scale bar: 50 μm

(g-i). Majority of MBP^+ OLs do not express GPR56 (j-l, red). Only ~ 10% MBP^+ OLs are $GPR56^+$ (m-o). Scale bars: 25 μm (a-f, j-o).

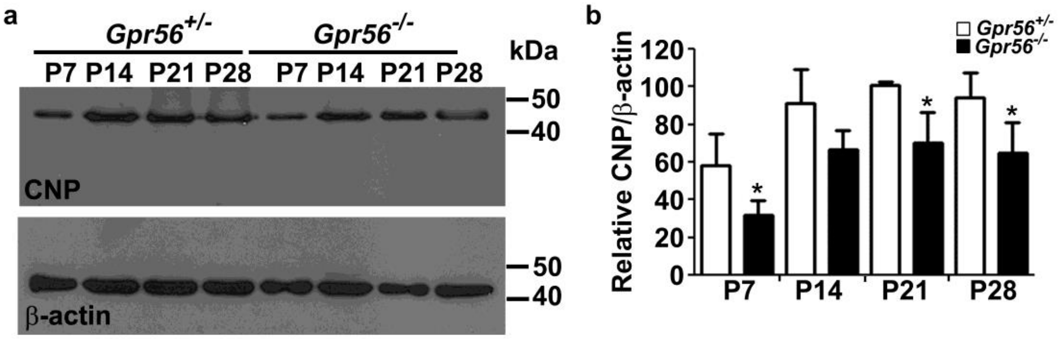

Supplementary Figure 6: CNP protein levels are reduced in the CC of *Gpr56*$^{-/-}$ mice. (a) Western blot detecting CNP protein level in the CC of *Gpr56*$^{+/-}$ and *Gpr56*$^{-/-}$ mice from P7 to P28. Loading control: β-actin. (b) The relative levels of CNP protein compared to the loading control were presented at P7 (*, p=0.0286), P14 (ns, p=0.0568), P21 (*, p=0.0229) and P28 (*, p=0.0276). Unpaired t-test, n = 4 per genotype. Error bars are means ± s.e.m.

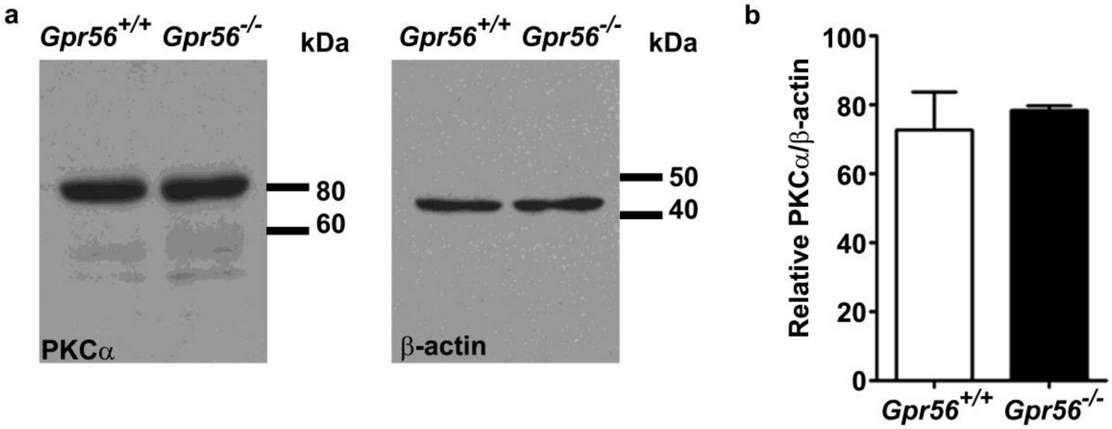

Supplementary Figure. 7: Loss of GPR56 has no effect on PKCα.

(a) Western blot of PKCα of P7 optic nerves of *Gpr56*$^{+/+}$ and *Gpr56*$^{-/-}$ mice. (b) The relative levels of PKCα were shown. p=0.6401; unpaired t-test, n = 3 per genotype. Loading control: β-actin. Error bars are means ± s.e.m.
